# Hot Blanket Packs in the Treatment of Fevers

**Published:** 1892-09

**Authors:** 


### HOT BLANKET PACKS IN THE TREATMENT OF FEVERS.

(*New York Medical Journal*, May 28, 1892, p. 606) Dr. W. W. Bremner describes in detail his method of reducing temperature in the treatment of typhoid fever and other febrile diseases. He employs a blanket just large enough to completely envelope the patient, folded lengthwise twice and then rolled up into a moderately tight roll. A boiling solution of soap, made by dissolving two ounces of good soap in two quarts of water, is then poured slowly into the centre of each end of the roll of blanket, so as to thoroughly saturate it. A bed is then prepared by covering it with a Mackintosh sheet, over which is laid a large, dry, double blanket. The patient should be undressed and have a loose blanket thrown over him until he can be enveloped in the hot blanket and the dry one wrapped around him. The arms are enclosed on both sides, first with a wet blanket, and then with a double dry one, care being taken to make the latter fit closely around the neck. If the feet are cold, a hot-water bottle should be applied to them. The pack should be continued from one to two hours, according to the state of temperature and the feelings of the patient. Children often fall asleep during the application, and seem rather to enjoy the procedure. After the blankets are removed, the patient should be gently rubbed with a soft towel and replaced in the ordi-dinary bed. The pack may be repeated two or three times a day, or as often as necessary until the temperature remains permanently below the danger point. Cold applications may be made to the head, and the patient may be supplied with cold water to drink as often as he wishes. The writer believes that this method of treatment has all the advantages of cold water applications without any of their drawbacks, and that the temperature can be lowered by a process of evaporation in this way just as certainly as by the use of cold water, so highly recommended by the followers of Brandt.—*Inter. Med. Mag.*

